# Correspondence

**Published:** 1890-01

**Authors:** C. D. Roy

**Affiliations:** Charity Hospital, New York


### Correspondence.

NEW YORK, December 4th, 1889.

New York is the mecca where not only the old and young practitioners gather to refresh themselves in the varied fields of medicine, but where hundreds of young men yearly come to seek an entrance into the mysteries of Æsculapius. It is thus being *in medias medicas* that I thought it would be of interest to the readers of THE RECORD to know something of the metropolitan city in that aspect which pertains to her medical nature. To him who seeks the study of medicine with a mind unbiased by the empiricisms of the ancients and the dogmas of a few, but with a mind ever seeking to add new thoughts and new deeds to their growing science, New York presents an enticing field of labor, which does not always prove "love's labor lost." For nowhere could a man wish for better or more varied clinical advantages, and no city could give him a better opportunity to master the intricacies of his profession than is offered in this place.

It is a pleasure for the South to know that some of the men who stand at the head of the medical profession in this city were nurtured upon her bosom, and gained their first knowledge from her Pyrean spring. Thomas, Wyeth, Sayre, Emmet are all Southerners, and there are many others here from the same clime, who are recognized as men of great ability.

The several medical colleges have reopened with seemingly renewed vigor, and the number of students who have matriculated are in excess of all previous years. The College of Physicians and Surgeons takes the lead, with seven hundred and twenty matriculants.

I have often thought whether these hundreds of young physicians who are annually turned at large will all of them follow this profession which they have chosen. How few men who study law ever spend their days as barristers.

Being at present connected with Charity Hospital, I have many opportunities of seeing tried the various advancements which have been made in the medical science, and coupled with the many interesting cases which are seen, I hope to give occasionally to THE RECORD those data which come under my observation.

A post-mortem performed by Dr. Biggs a few days ago at the hospital presented a very peculiar and yet a very unique state of affairs. The patient, a man of fifty years, had died of phthisis, as was confirmed by the symptoms during life and the post-mortem appearances. The point in question refers to the lungs, which, besides showing the usual appearances of phthisical lungs, had projecting out from the apex of the right lobe a spicula of real bone, and scattered throughout the substance of the left lobe were various other smaller spiculæ. Dr. Biggs said that it was the second case he had met with, and as far as he knew the third case on record. It could only be explained by the fact of extensive adhesions between the chest wall, pleura, and lung tissue, in which the osteogenetic layer of the bones had imbedded itself into the lung tissue by the prolific-ration of its cells.

A great method of treating phthisis in the hospital, and one which has shown some excellent results, is the inhalation method. Not that of heated air, or vapor, which, to be of a sufficient temperature to kill the germ, must at the same time do injury to the bronchial tract, but that of carbolic acid or creasote. This remedy, if not of sufficent germicidal power to kill the micro-organisms, has certainly proven, in the hands of the physicians here, to be able to hinder the germs in their ravages upon the patients. Besides the usual remedies of cod liver oil and iron in the weak and anæmic, an inhalation mixture is made, and the patient made to inhale it for half an hour three times a day. The mixture is as follows:

℞ Ol. eucalypti, } aa. ʒi

Ol. creosoti, }

Ol. Terebentheræ ʒriij.

M. Sig. One teaspoonful in an inhaler three times a day.

Before closing I wish to say something of a test for sugar in the urine, which is one of fast-growing popularity in the profession. The test, which is known as Alexander's, is the one much used by the Germans, and is found in all the German text-books, but it has only lately been used to any extent by our brothers on this side of the water. The test fluid is made as follows:

Take 200 parts of a ten per cent. solution of caustic soda, and add to that eight (8) parts of Rochelle salts, and dissolve the same by heating. To this solution, while still hot, add four (4) parts of sub-nitrate of bismuth. Keep this prepared preparation in a colored bottle. When you wish to test the urine, add one-half as much urine as you have of the solution in the test-tube, and then heat. As the heating progresses the fluid, if there be sugar present, begins to darken, and may reach a very dark brown color.

The writer has noted with pleasure the presence in the city of Drs. Cooper and James Avary, of Atlanta.

C. D. ROY, M. D.

*Charity Hospital, New York.*

